# Promoting the use of a self-management strategy among novice chiropractors treating individuals with spine pain: A mixed methods pilot clustered-clinical trial

**DOI:** 10.1371/journal.pone.0262825

**Published:** 2022-01-21

**Authors:** Owis Eilayyan, Aliki Thomas, Marie-Christine Hallé, Anthony C. Tibbles, Craig Jacobs, Sara Ahmed, Michael J. Schneider, Fadi Al Zoubi, Joyce Lee, Danny Myrtos, Cynthia R. Long, Andre Bussieres

**Affiliations:** 1 School of Physical and Occupational Therapy, McGill University, Quebec, Canada; 2 Physical Therapy and Rehabilitation Department, College of Applied Medical Sciences, Jouf University, Sakaka, Jouf, Saudi Arabia; 3 Center for Interdisciplinary Research in Rehabilitation of Greater Montreal (CRIR), Quebec, Canada; 4 Canadian Memorial Chiropractic College, Ontario, Canada; 5 School of Health and Rehabilitation Sciences, University of Pittsburgh, Pittsburgh, Pennsylvania, United States of America; 6 Department of Rehabilitation Sciences, The Hong Kong Polytechnic University, Hung Hom, Hong Kong; 7 Palmer College of Chiropractic, Davenport, Iowa, United States of America; Prince Sattam Bin Abdulaziz University, College of Applied Medical Sciences, SAUDI ARABIA

## Abstract

**Background:**

The uptake of Self-Management Support (SMS) among clinicians is suboptimal. To date, few studies have tested knowledge translation (KT) interventions to increase the application of SMS in chiropractic teaching clinics.

**Study objective:**

Evaluate the feasibility of implementing a KT intervention to promote the use of a SMS strategy among chiropractic interns, their supervisors, and individuals with spine pain compared to controls.

**Methods:**

Mixed methods pilot clustered-clinical trial. Clusters of 16 Patient Management Teams were allocated to a complex KT intervention (online and workshop training). Primary feasibility outcomes for clinicians, interns and patients were rates of recruitment, retention, and adherence to protocol. A nominal group technique and interviews were used to seek end-users’ views on the implementation process, and generate possible solutions.

**Results:**

In total, 16 (84%) clinicians, 65 (26%) interns and 42 patients agreed to participate. All clinicians in the intervention group completed all KT intervention components, 23 interns (85%) completed the online training and 14 interns (51.8%) attended the workshop training. All clinicians in the intervention and seven (78%) in the control group completed all outcome measures at baseline and 6-month follow-up, while 15 (55.6%) and 23 (60.5%) interns in the intervention and control groups completed the questionnaires at baseline and 6-month follow-up, respectively. Among patients, 10 (52.6%) and 12 (52.2%) in the intervention and control groups respectively completed the questionnaires at the end of the study. Based on interview findings, solutions to improve the feasibility of conducting a full trial include: making SMS a part of the internship, changing the time of introducing the study to the interns, and having more training on SMS.

**Conclusion:**

Recruitment and retention of chiropractic interns and patients for a larger implementation trial in a single outpatient teaching clinic may be challenging.

## Introduction

Spine pain is a leading cause of disability worldwide [1,2]. For many, spine pain is a long-term condition leading to physical, psychological, and emotional burden [3,4]. Nearly half of the individuals with low back pain seek relief from healthcare providers [5], and many choose to consult chiropractic interns and their supervisory clinicians in outpatient teaching clinics such as at the Canadian Memorial Chiropractic College (CMCC), in Ontario, Canada [6].

Recent clinical practice guidelines (CPGs) recommend multimodal care, including patient education and advice about Self-management support (SMS) strategies [7–10]. SMS aimed at enabling people to adopt a healthy lifestyle [11,12] are key to effectively manage pain and related co-morbidities [3,13–19], and reduce the associated burden [20]. SMS is defined as an “individual’s ability to manage the symptom, treatment, physical and psychosocial consequences and lifestyle changes inherent in living with a chronic condition, P.129” [21]. SMS contributes to reducing pain and psychological distress and improving functional ability for patients with spine pain [22,23]. However, routine use of evidence-based practice (EBP) and CPGs that recommend the use of SMS remains suboptimal among chiropractors [24–27]. Furthermore, patient engagement in SMS programs is also suboptimal [28–30].

Changing clinician and patient behaviour is challenging [25,31]. SMS strategies require patients to change unhealthy behaviours and commit to healthier ones [11,12]. Also, clinicians may fear the consequences of shifting away from traditional practices [32]. In related studies, we identified barriers to the routine use of SMS among CMCC clinicians who are supervising chiropractic interns [27], and patients seeking care for spine pain [33]. Clinician and intern barriers included lack of skills, knowledge, and time; anxiety toward using SMS; and the supervisory clinicians’ perception of SMS restricted their use of SMS [27]. Patient-related barriers included lack of time and educational materials, anxiety about performing the exercises without pain and difficulty in remembering SMS components [33]. Other factors reported in the literature include low self-efficacy, disbeliefs in SMS, family commitments, and depression [32,34,35].

Knowledge Translation (KT) is a process used to promote the uptake and application of best practices by changing professional, patient, and decision-maker behaviours [36,37]. Current evidence supports the use of multilevel (targeting both clinicians and patients) and multifaceted (combining several elements) KT interventions [38]. Theory-based KT interventions aim to simultaneously address clinician specific barriers [39], patients’ adherence to clinical recommendations, increase clinicians’ use of best evidence, and optimise patient health outcomes [38].

There is evidence suggesting that promoting the use of EBP and CPGs early during professional training may be more effective than changing the practice of seasoned clinicians who are less likely to change the way they “always do things” [40,41]. Academic programs play a vital role in helping shape future health providers’ EBP competencies; they must first lay the foundations of EBP (the “what”, “why”, “how” and “when” of EBP) and, over the course of training, ensure that students move along a trajectory of progressive development of EBP competencies [40,42]. Importantly, educational institutions are expected to adequately prepare future graduates to appreciate and navigate the many organizational factors (e.g., patient overload, staff shortage) that can often impede the sustained use of EBPs [43]. In the context of becoming evidence-based practitioners, Singer and Bossarte (2006) suggest three reasons for why students may be interested in taking part in a research project during their formal training: 1) altruistic purposes” as people want to help others; 2) project-related purposes” (e.g., interesting topic); and 3) “self-serving purposes” (e.g., incentives) [44].

Incorporating EBP in the formal teaching help interns become lifelong learners and reflective practitioners, which can overcome barriers to the uptake of CPGs and reduce the gap between EBP and actual practice [25,45]. Health professions education that successfully promotes the use of CPGs in the management of spine pain has the potential to better prepare graduates to embrace and apply best practices.

To date however, few studies have tested the implementation of innovative multicomponent KT interventions aimed at changing chiropractors’ and interns’ practices. As costly randomised clinical trials (RCTs) are only justified once feasibility is established and the implementation protocol is refined [46], the primary objective of this pilot study was to evaluate the feasibility of conducting a full implementation trial of a KT intervention promoting the use of SMS among chiropractic interns seeing patients with spine pain compared to controls. Feasibility was evaluated in terms of rates of recruitment, retention, and adherence to the study protocol. The secondary objectives were to estimate 1) the potential effectiveness of a KT intervention on SMS skills and confidence among supervisory clinicians and interns; and 2) self-care activation [the knowledge and confidence of self-management [47] among patients with spine pain compared to the control group.

## Methods and material

**Fig 1:**
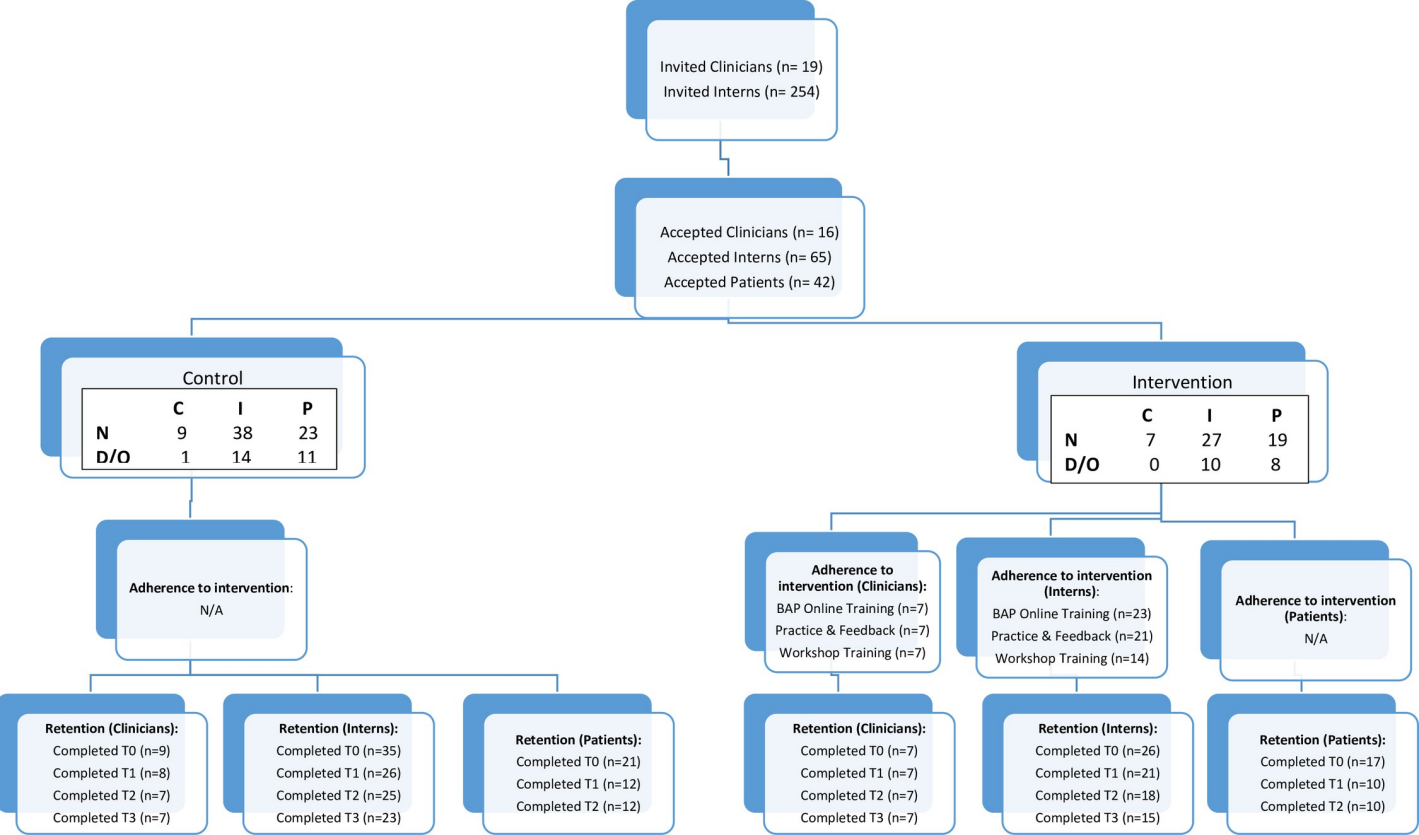
Participant recruitment flowchart. C: Clinicians, D/O: Dropped-out, I: Interns, N: Number, P: Patients.

This study was reported according to the CONSORT Extension to Pilot and Feasibility Trials checklist and flow diagram [48] Fig 1. The authors confirm that all ongoing and related trials for this intervention are registered (ISRCTN17077842). This study was registered after the enrolment of participants began, as the corresponding author was not aware that this was a required procedure for pilot implementation trials.

*Design*: We conducted a mixed methods pilot clustered-clinical trial comparing two methods of delivering an educational intervention: (1) a complex KT intervention plus dissemination of the Brief Action Planning (BAP) flowchart for the intervention group; and (2) passive dissemination of the BAP flowchart alone for the control group. The rates of recruitment, retention, and adherence to the protocol were the primary feasibility outcomes for clinicians, interns and patients. In addition, a nominal group technique and interviews were used to seek end-users’ views on the implementation process, and generate possible solutions.

*Study context*: This was a multi-phase study aimed at promoting the use of SMS among supervisory clinicians and interns treating individuals with spine pain in three outpatient chiropractic-teaching clinics over a 24-month period. These clinics are affiliated with a chiropractic teaching institution in Toronto, Ontario (CMCC). Across the 3 participating clinics, 16 Patient Management Teams (PMTs) deliver patient care. Each PMT is composed of one supervisory chiropractic clinician who supervises 7–9 chiropractic interns. The interns are responsible for managing patients during their 12-month internship under the direct supervision of the chiropractor.

Ethical approval was obtained from the Research Ethics Board of McGill University (McGill IRB: A09-B53-17B), and from the Research Ethics Board of CMCC (1711X01). All participants signed an informed consent form.

### Participants and procedures

Twenty CMCC supervisory clinicians and 255 interns from three outpatient clinics were first informed of the study purpose and encouraged to participate in a meeting led by the Dean of the CMCC clinics (AT). Potential participants received an ethics approved study summary. All interested clinicians and interns received a follow-up letter with additional information about the study. A recruitment notice was also posted in the waiting rooms to inform patients of the study. Each participating intern was asked to recruit up to three consecutive patients seeking care for spinal pain. The research coordinator and participating clinicians and interns solicited potential patients to participate using an ethics approved letter. To avoid participant coercion, a research coordinator delivered the informed consent process in a private room without the presence of clinicians or interns.

*Clinicians*: Supervising clinicians were eligible to participate if they: 1) were licenced chiropractors; and 2) worked either part time or full-time at one of three CMCC outpatient clinics.

*Interns*: The interns were eligible to participate if they: 1) were in their final year at CMCC; 2) worked in one of 16 PMTs who agreed to participate in the study; and 3) provided chiropractic treatment to a least two adults (age 18–65) with spine pain each week. Interns were excluded from the study if they had already attended the BAP webinar or educational online module. Prior online registration provided the mechanism for confirming study eligibility.

*Patients*: Patients were deemed eligible if they were: an adult between the ages of 18 and 65; **had non-specific back pain**, received treatment by a consenting chiropractic intern; and were able to read English and hold a conversation in English. Patients were excluded if: 1) their clinical file revealed the presence of a ‘red flag’ (i.e., indicators of serious pathologies including malignancy, infection, fracture, inflammatory disease); and 2) were pregnant, which might lead to reduced adherence due to possible attrition.

### Design of the KT intervention

Informed by a systematic approach proposed by French et al (2012) [49], our team adapted a theory-based KT intervention previously developed for a related project [50]. Regarding the first question of this framework (“*Who needs to do what*, *differently*?” *i*.*e*., *identify the evidence-practice gap*), the literature suggests that the use of SMS among clinicians and patients is suboptimal [32,34,35]. The second and third questions (“*Which barriers and enablers need to be addressed*” *and* “*which intervention components (behaviour change techniques and mode(s) of delivery) could overcome the modifiable barriers and enhance the enablers*?”) were addressed in two related projects [27,33]. Briefly, three focus groups of clinicians and interns, and 13 online individual interviews of spine pain patients were conducted based on the Theoretical Domains Framework (TDF) [51] to identify barriers and enablers to the use of SMS. Findings informed the design of the KT intervention by mapping key barriers to behaviour change techniques, using a panel of experts. The results reported in this paper address the fourth question “*How can behaviour change be measured and understood*?*”*

### Intervention description

**Supervising chiropractors:** Prior to the study onset, PMT supervisory clinicians allocated to the theory-based KT intervention received a 50-minute webinar, a 22-minute online educational module on the BAP, and educational materials (BAP flowchart, poster) to promote understanding of the BAP, memory of BAP and use of the BAP. Two BAP trained/certified clinicians took the role of facilitators/opinion leaders; they advised colleagues on the SMS practice, eased the delivery of SMS, and facilitated completion of study questionnaires. Three months later, the supervising clinicians in the intervention group attended a one-day training session provided by a certified BAP trainer. The training served as a reminder of using BAP properly and provided clinicians with more in-depth knowledge of SMS using BAP. They also had the opportunity to practice the BAP and further improve their SMS skills using role-plays.

The BAP framework was used to guide the SMS in this study. It is a structured tool developed based on motivational interviewing and used to promote the implementation of self-management for patients with chronic conditions [52]. Briefly, the BAP utilizes a stepwise process where clinicians work collaboratively with patients to ensure goals and action plans are meaningful and realistic. It includes three questions and five skills including “offering a behavioural menu, SMART planning, eliciting a commitment statement, problem solving for low confidence, and follow up” [52] (S1 Appendix). The BAP framework can be used to enable the use of SMS in different clinical settings and training programs [52,53], and may be considered an ideal SMS program in busy clinics [52].

**Interns:** At study onset, consenting interns accessed the 50-minute BAP webinar, the 22-minute online educational module on the BAP and educational material (BAP flowchart) during the 5-hour weekly block dedicated to administrative duties. They were also provided with a demonstration on how to effectively use the BAP during patient clinical encounters, along with practice and feedback opportunities. They also had the opportunity to practice the BAP in role-play situations to further improve their skills of SMS. Then, the interns in the intervention group received additional BAP skills training from their clinicians supported by certified opinion leaders.

**Patients:** Individuals with back pain in the intervention group seeking care from trained interns and supervisory clinicians received handouts summarising and demonstrating the therapeutic recommendations related to SMS (e.g., home exercise and other lifestyle changes). In addition, they were engaged in the BAP process led by BAP trained interns.

**Control group. Supervising chiropractors:** PMT supervisory clinicians in the control group only received a copy of the guidelines on managing spine pain [9,54] and a BAP flowchart.

**Interns:** Interns in the control group received only a copy of the guidelines on managing spine pain [9,54] and a BAP flowchart. At six months (i.e., after the end of the data collection), the interns in the control group were also exposed to the online training of the KT intervention (BAP webinar and educational module).

**Patients:** Individuals in the control group participated in the BAP process led by interns in the control group.

#### Allocation to intervention methods

The onsite research coordinator assigned consenting PMT supervisory clinicians and interns to one of two groups based on working days: Group 1 (intervention) workdays = Tuesday, Thursday, Saturday, and Group 2 (control) workdays = Monday, Wednesday, Friday. There was no overlap in the schedule between clinicians and interns (i.e., clinicians and interns only work three days per week on these specific days), and there was no difference in demographic characteristics, between clinicians, interns, and patients among both groups. To avoid contamination, the clinicians and interns in the intervention group were asked not to share the educational materials with colleagues in the control group.

#### Concealment of the allocation sequence

Clinicians and interns were informed of the study groups after allocation.

#### Blinding

Investigators (not involved in the delivery of the intervention) and patients were blinded to group allocation until the statistical analyses were completed. Interns and clinicians were aware of the KT interventions they were receiving and/or implementing. They were not explicitly informed of their assigned groups and study hypotheses.

### Data collection and management

*Recruitment rate*: Obtained by calculating the number of participating interns and supervisory clinicians over the number of potentially eligible participants.

*Retention*: Follow-up in terms of both attended appointments and completion of questionnaires, was assessed at the end of the study.

*Adherence*: Measured by the rate of completion of the webinar, completion of 2 clinical vignettes and the self-management learning module.

*Intervention fidelity*: Interns and supervisory clinicians completing the BAP skills survey and BAP Tool Experience (S2 Appendix) at four time-points during the study: 1) at the start of the study, 2) immediately after the online training, 3) immediately after the workshop training, and 4) at the end of the study. To monitor if interns were using the BAP in accordance with its principles (fidelity assessment), an observer reviewed the interaction with a real patient using the BAP skills checklist (S3 Appendix).

*Patient data*: We assessed: 1) the “patient’s activation”, defined as an individual’s knowledge, skill, and confidence for managing their health and health care [55], 2) level of confidence with SMS compliance with their action plan, 3) pain intensity, 4) functional disability, and 5) satisfaction with care at the start of the study, at week 2, and at the last treatment session or week 8. Data were collected and stored on LimeSurvey.

### Mid-point Nominal Group Techniques (NGTs) (Fig 2) & end of study individual interviews

Six months after implementing the KT intervention, one NGT [56] session was conducted with decision makers, clinicians and interns, and another with patients to obtain their views about the challenges encountered in the implementation of SMS, and to help generate possible solutions for a larger trial. The participants in the NGT were first asked to generate a list of challenges encountered during the study. Next, they elaborated on the challenges and ranked them from the most to least important. At the end of the NGT, the participants suggested solutions to address the challenges and enhance the conduct of the study (e.g., refining recruitment procedure and intervention implementation process) in view of the future potential full trial (e.g., randomization and evaluation tool). The NGT session lasted approximately 2 hours.

**Fig 2:**
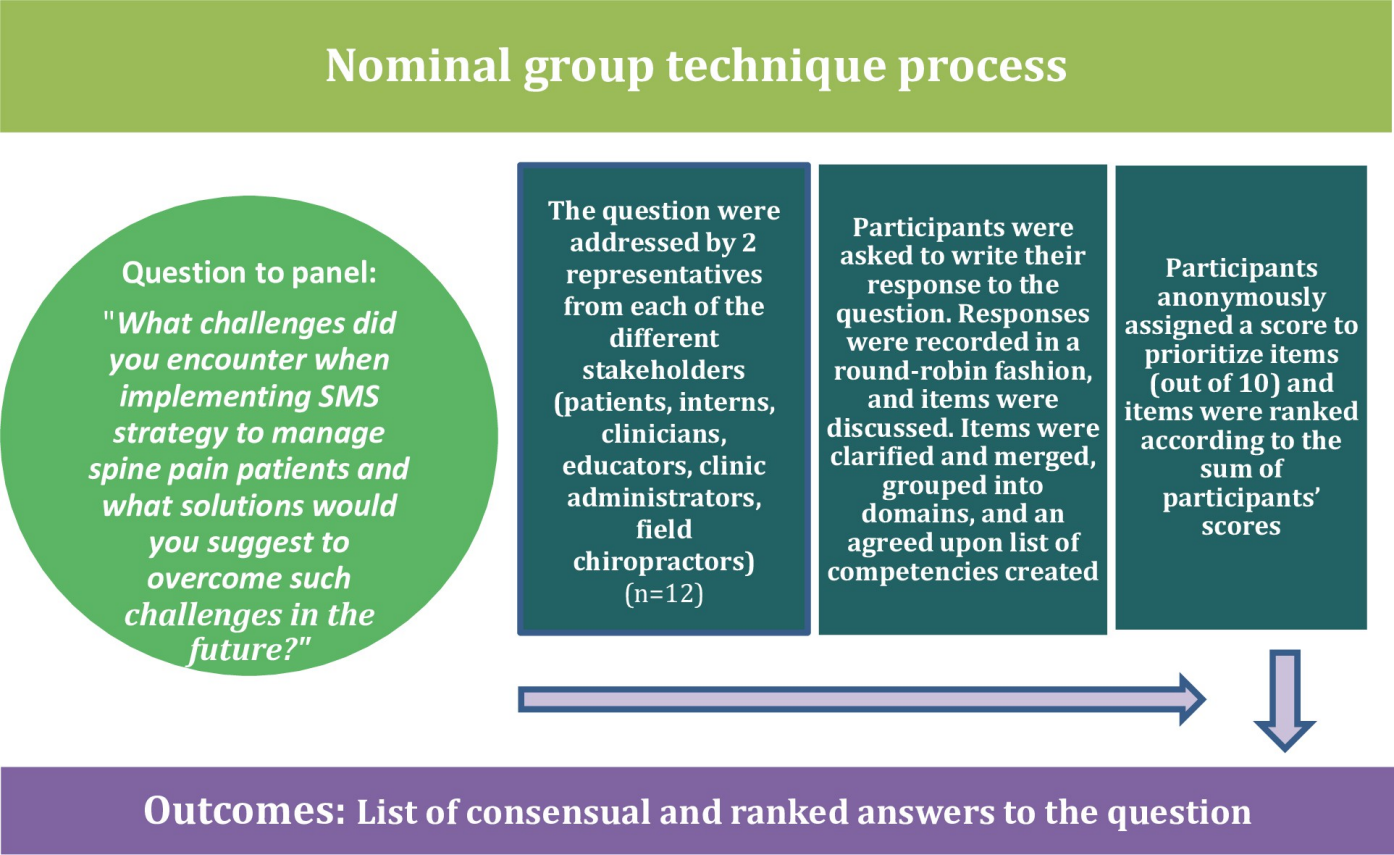
Nominal Group Technique used to identify implementation challenges and possible solutions.

We also conducted end of study individual interviews with clinicians, participating interns, interns who dropped-out, non-participating interns, and patients. The aims were to: 1) discuss the challenges encountered in the implementation of SMS; 2) explore possible considerations for leading a larger trial; and 3) identify the reasons why interns decided to withdraw from, or not participate in this study. Each interview lasted approximately 20 minutes.

### Outcomes measures

Primary feasibility outcomes for clinicians, interns and patients were recruitment rates, retention rates and adherence to the KT intervention. S4 Appendix provides a description of the feasibility outcomes, sources of measurement, and timing of administration. S5 Appendix reports the criteria to assess feasibility to support moving to a full-scale C-RCT.

#### Criteria to assess feasibility

For supervising clinicians and interns, acceptable feasibility criteria were defined as: 55% of eligible clinicians and interns would agree to participate (i.e., recruitment rate); of these, 90% would complete the intervention components (adherence rate), and 80% would complete the study at 6 months (i.e., retention rate). For patients, acceptable feasibility criteria were defined as: three patients per intern enrolled within 6 weeks of recruitment notice (i.e., recruitment rate), and 80% completion of patient encounter forms at baseline and at follow-up at 8 weeks (i.e., retention rate) (S5 Appendix).

#### Clinician and intern outcomes

Adherence to the intervention protocol was calculated as the number of interns adhering to the intervention protocol per the total number of participants. Perceptions regarding skills for using PAB were assessed using the BAP skills tool consisting of 10 items rated on a 4-point scale. Higher scores reflect higher confidence regarding one’s perception of their skills in using BAP. Self-efficacy and perceived importance related to the BAP measured by the tool experience survey. It included two subscales; BAP self-efficacy and perceived importance, each containing 7 items rated on a 10-point scale. Higher scores reflect higher confidence regarding perceived self-efficacy and importance related to the BAP.

#### Patient outcomes

Accurate and timely completion of questionnaires (BAP survey, Patient Activation Measure, Numerical Rating Scale, Bournemouth Questionnaire, PROMIS Global Health Questionnaire, patients’ satisfaction, and extent of a “patient’s activation” that was measured by Patient Activation Measure (PAM)) [55]. The PAM includes 13-items on the knowledge of, and confidence in one’s involvement in self-care [47]. The scores range from 0–100, where higher score indicates better outcome. Patient secondary outcomes included level of confidence with SMS compliance with their action plan, risk of delayed recovery (STarT Back Screening Tool) [57], pain intensity measured by Numerical Rating Scale (NRS) [58], disability measured by Bournemouth Questionnaire (BQ) where the higher indicates the worse outcome [59], quality of life measured by PROMIS Global Health questionnaire) [60,61], and satisfaction with care.

### Data analysis

#### Quantitative data

The main analysis was descriptive statistics to characterize participant feasibility measures (recruitment rates, retention rates, and adherence to the intervention). Secondary analyses were used to estimate interns’ perceived skills, self-efficacy and perceived importance related to the BAP; and patient outcomes of pain, disability, and activation. All statistical analysis was done using SAS 9.4 [62]. Due to the high drop-out rate (39.5%), we were unable to estimate key parameters such as effect sizes for a future full-scale cluster RCT, or patient adherence to the recommended SMS approach for spine pain.

The statistical analysis of the primary study endpoint was based on an intention to treat analysis (i.e., all participants were analysed as allocated). Non-parametric tests were used to assess the effectiveness of the intervention on clinicians’ outcomes as the assumptions of t-test were not met; Wilcoxon-Mann-Whitney test (between groups) and Wilcoxon signed rank sum test (within group). The multiple comparisons adjustment method was not applied.

As the interns were the unit of analysis, generalized linear mixed effect models were used to consider the effect of clinician groups. Time was considered as the fixed effect, to assess the effectiveness of the intervention on interns’ outcomes at the cluster level. The assumptions of the model were checked, and the convergence criteria met. Degrees of freedom was calculated using the Satterthwaite method. Those with missing data at each induvial time point were not used in the analysis. S6 Appendix showed the statistical analyses syntax.

#### Qualitative data

Data from the NGT and individual interviews were anonymized and transcribed verbatim by one of the research team members (OE). We conducted an inductive thematic analysis [63], whereby we 1) read and familiarized ourselves with the raw textual data (i.e. transcriptions); 2) generated a list of initial codes by establishing clear links between the exploratory nature of our research objectives and our reading of the raw data; 3) generated overarching themes regarding the challenges reported by the participants; and 4) mapped practical solutions to challenges identified, based on the experiences that we identified in the data. Findings aimed to inform adaptation/improvement of the study protocol prior to undertaking a future large cluster RCT.

## Results

Sixteen supervisory clinicians, 65 interns and 42 patients participated in the study. There were no differences in the baseline characteristics among clinicians and interns in the intervention and control groups. The mean age of clinicians in the intervention and control groups was 47.5 ± 10.5 and 54.3 ± 10.3 years, respectively. The mean age of interns in the intervention and control groups was 27.15 ± 3.1 and 27.13 ±2.6 years, respectively. The baseline characteristics of participants are shown in Tables 1 and 2.

**Table 1:** Baseline characteristics of supervisory clinicians.

	Intervention (N = 7)	Control (N = 9)
Age (Yrs): Mean (SD)	47.5 (10.5)	54.3 (10.3)
Gender (% Women)	3 (43%)	1 (11%)
SMS Knowledge (% Yes)	5 (71%)	9 (100%)
BAP Knowledge (% No)	7 (100%)	9 (100%)
SMS Skills: M (SD) / (1–4)	1.6 (0.38)	2.2 (0.79)
SMS Confidence: M (SD) / (0–10)	7.1 (1)	7.8 (0.73)
SMS Importance: M (SD) / (0–10)	8.6 (0.79)	8.7 (0.74)

**Table 2:** Baseline characteristics of interns.

	Intervention (N = 23)	Control (N = 35)
Age (Yrs) Mean (SD)	27.26 (3.1)	27.19 (2.6)
Gender (% Women)	14 (60.87%)	14 (36.84%)
SMS Skills: M (SD) / (1–4)	1.7 (0.58)	1.8 (0.56)
SMS Confidence: M (SD) / (0–10)	6.75 (1.3)	6.7 (0.99)
SMS Importance: M (SD) / (0–10)	9.2 (0.91)	9.2 (0.62)

The mean age of patients in the intervention and control groups was 35.7 ± 13.6 and 50.8 ± 13.2 years, respectively. The majority of participants in the intervention were female (70.6%), while in the control group, 47.6% were female. Baseline characteristics differed between patients in the intervention and control groups for age, confidence in BAP, NRS, BQ, PROMIS-Mental, and Keele STarT Back patient-reported outcomes (Table 3).

**Table 3:** Baseline characteristics of patients.

	Intervention (17)	Control (21)	p-value
Age (yrs) Mean (SD)	35.77 (13.6)	50.81 (13.24)	0.003
Gender (female %)	70.59%	47.62%	0.2
Marital Status: n (%)			0.04
Never Married	10 (58.8%)	7 (33.3%)	
Married	7 (41.2%)	8 (38.1%)	
Divorced	0 (0%)	6 (28.6%)	
Other Complaints			0.73
Yes	12 (70.59%)	16 (76.19%)	
No	5 (29.41%)	5 (23.81%)	
BAM Level			0.47
Level 1	3 (17.65%)	5 (25%)	
Level 2	2 (11.76%)	6 (30%)	
Level 3	9 (52.94%)	7 (35%)	
Level 4	3 (17.65%)	2 (10%)	
PAM Score	62.43 (12.3)	55.24 (11.58)	0.08
BAP Confidence	7.76 (1.75)	5.33 (2.89)	0.01
BAP Score	2.84 (1.1)	3.08 (1.22)	0.64
NRS	2.59 (1.7)	4.81 (2.9)	0.01
BBQ	20.53	32.55 (14.21)	0.02
PROMIS Physical	41.05 (3.99)	44.35 (6.72)	0.13
PROMIS Mental	47.45 (7.18)	38.88 (6.64)	0.002
Total Keel Test	2.41 (1.54)	4.11 (2.3)	0.03

### Supervising clinicians

#### Recruitment rate

Of the 20 supervisory clinicians invited to participate in the study, 16 agreed to participate (recruitment rate of 80%). Seven and nine clinicians were assigned to the intervention and control groups, respectively. One clinician from the control group withdrew from the study as none of his interns participated in this study.

#### Adherence to the KT intervention

All clinicians in the intervention group (100%) completed the BAP online training and practice and feedback session, attended the workshop training, and received a certification on BAP.

#### Retention rate

All seven clinicians in the intervention group (100%), and 77.8% (7/9) in the control group (including the withdrawals) completed and returned questionnaires at 6 months (i.e., end of the study questionnaires).

#### Perceived BAP skills, self-efficacy and importance

*Within group*. There was a difference among clinicians in the intervention group regarding the changes in skills immediately after the online training intervention (change score among intervention group = 1.1; 95% CI = 0.5–1.5, *P* = 0.03), after the workshop training (change score = 1.22; 95% CI = -0.3–2.4, *P* = 0.03), and at the mid-point of the study (change score = 1.48; 95% CI = 0.7–1.8, *P* = 0.03). There was no difference in any other measure for the intervention and control groups (Table 4).

**Table 4:** Difference between clinicians in the intervention and control groups (*between groups)*.

Measure	Intervention	Control	Between group difference	95% CI	P-value
Skills pre-post	1.1 (0.88)	0.24 (0.68)	0.86	-0.3–1.4	0.03*
Confidence pre-post	0.51 (0.82)	-0.13 (0.53)	0.64	-0.2–1.3	0.04*
Importance pre-post	0.24 (0.77)	-0.39 (0.6)	0.63	-0.7–1.1	0.03*
Skills pre-mid point	1.48 (0.74)	0.38 (0.87)	1.1	0.1–1.7	0.06
Confidence pre-mid point	0.86 (0.89)	-0.02 (0.39)	0.88	-0.56–1.6	0.03*
Importance pre-mid point	0.33 (0.86)	-0.12 (0.46)	0.45	-0.8–0.6	0.18
Skills pre-end point	1.37 (0.85)	0.27 (0.92)	1.1	-0.3–2.4	0.046*
Confidence pre-end point	0.78 (0.85)	0.24 (0.59)	0.54	-0.57–1.57	0.11
Importance pre-end point	0.29 (0.77)	-0.06 (0.39)	0.35	-0.57–1	0.18

* Significant at 0.05, **pre-post**: Immediately after intervention, **pre-mid point**: 3 months after the intervention, **pre-end point**: 6 months after the intervention.

*Between groups*. There was a difference between clinicians in the intervention and control groups regarding the changes in skills (Mean Difference (MD) among intervention group = 1.1; controls = 0.24; 95% CI = -0.3–1.4, *P* = 0.03), confidence of implementing BAP (MD among intervention group = 0.51 vs controls = -0.13; 95% CI = -0.29–1.29, *P* = 0.04), and perceived importance of BAP (change score among intervention group = 0.24 vs controls = -0.39; 95% CI = -0.71–1.1, *P* = 0.03) immediately after the intervention (Table 5). Also, there was a difference between clinicians in the intervention and control groups regarding the changes in confidence of implementing BAP at the mid-point of the study (change score among intervention group = 0.86 vs controls = -0.02; *P* = 0.03) and in skills at the end of the study (change score among intervention group = 1.37 vs controls = 0.27; *P* = 0.046).

**Table 5:** Differences before and after among clinicians in the intervention and control group (*within groups)*.

Measure	Intervention			Control		
	Mean	95% CI	p-value	Mean	95% CI	P-value
Skills pre-post	1.1 (0.88)	0.4–1.8	0.03*	0.24 (0.68)	-0.23–0.7	1.0
Confidence pre-post	0.51 (0.82)	-0.1–1.12	0.45	-0.13 (0.53)	-0.5–0.24	0.73
Importance pre-post	0.24 (0.77)	-0.33–0.81	0.45	-0.39 (0.6)	-0.8–0.03	0.07
Skills pre-workshop	1.22 (0.78)	0.6–1.84	0.03*	-	-	-
Confidence pre- workshop	0.8 (0.9)	0.13–1.47	0.13	-	-	-
Importance pre- workshop	0.47 (0.63)	0.003–0.94	0.45	-	-	-
Skills pre-mid point	1.48 (0.74)	0.93–2.03	0.03*	0.38 (0.87)	-0.26–1	1.00
Confidence pre-mid point	0.86 (0.89)	0.2–1.52	0.13	-0.02 (0.39)	-0.31–0.27	1.0
Importance pre-mid point	0.33 (0.86)	- 0.31–0.97	0.69	-0.12 (0.46)	-0.46–0.22	0.69
Skills pre-end point	1.37 (0.85)	0.69–2.05	0.06	0.27 (0.92)	-0.39–0.97	1.00
Confidence pre-end point	0.78 (0.71)	0.25–1.31	0.45	0.24 (0.39)	-0.05–0.53	0.69
Importance pre-end point	0.29 (0.77)	-0.28–0.86	1.0	-0.06 (0.39)	-0.35–0.23	0.45

* Significant at 0.05, **pre-post**: Immediately after intervention, **pre-mid point**: 3 months after the intervention, **pre-end point**: 6 months after the intervention.

### Interns

#### Recruitment rate

Of the 254 interns invited to participate in the study, 65 agreed to participate (25.6% recruitment rate). Of the 27 and 38 interns assigned to the intervention and control groups respectively, 10 (37%) and 14 (37%) in each group withdrew from the study.

#### Adherence to the KT intervention

Of the 27 interns who were assigned to the intervention group, 23 (85%) completed the BAP online training, 21 (77.8%) attended the practice and feedback session, and 14 (51.8%) attended the workshop training.

#### Retention rate

Overall, 15 (55.6%) and 23 (60.5%) interns in the intervention and control groups completed end of the study questionnaires at 6 months, respectively.

#### Perceived BAP skills, self-efficacy and importance

The mixed-effect models showed that perceived BAP skills, self-efficacy and importance increased over time among interns in both groups, while there was no effect of the intervention components on the aforementioned outcomes. However, the interaction term of time and group showed that KT intervention increased the perceived BAP skills for interns in the intervention group at the mid- and end-points of the study (Tables 6–8).

**Table 6:** Mixed-effect model output (outcome = BAP skills).

Factor		Estimate	CI 95%	P-value
Age		0.01	-0.03–0.05	0.64
Gender	Female (ref)	-		-
	Male	0.23	-0.03–0.49	0.09
Group	Intervention (ref)	-		-
	Control	0.11	-0.2–0.42	0.49
Time	T0 (ref)	-		-
	T1	0.58	0.3–0.86	< 0.0001
	T2	1.02	0.74–1.3	< 0.0001
	T3	1.42	1.12–1.72	< 0.0001
Group * Time	T1*Intervention (ref)	-		-
	T1*Control	-0.16	-0.52–0.2	0.38
	T2*Intervention (ref)	-		-
	T2*Control	-0.38	-0.75 –-0.01	0.044
	T3*Intervention (ref)	-		-
	T3*Control	-0.49	-0.81 - -0.17	0.012

SMS: Self-management support, T0: Baseline, T1: Immediately after intervention, T2: Mid-point (after 3 months), T3: End-point (after 6 months).

**Table 7:** Mixed-effect model output (outcome = importance of SMS).

Factor		Estimate	CI 95%	P-value
Age		-0.04	-0.095–0.015	0.16
Gender	Female (ref)	-		-
	Male	0.1	-0.21–0.41	0.51
Group	Intervention (ref)	-		-
	Control	0.06	-0.3–0.42	0.76
Time	T0 (ref)	-		-
	T1	-0.01	-0.29–0.27	0.94
	T2	0.32	0.03–0.61	0.03
	T3	0.38	0.08–0.68	0.01
Group * Time	T1*Intervention (ref)	-		-
	T1*Control	0.17	-0.2–0.54	0.36
	T2*Intervention (ref)	-		-
	T2*Control	-0.26	-0.64–0.12	0.18
	T3*Intervention (ref)	-		-
	T3*Control	-0.21	-0.6–0.18	0.29

CI: Confidence interval, SMS: Self-management support, T0: Baseline, T1: Immediately after intervention, T2: Mid-point (after 3 months), T3: End-point (after 6 months).

**Table 8:** Mixed-effect model output (confidence to use SMS).

Factor		Estimate	CI 95%	P-value
Age		-0.001	-0.08–0.08	0.98
Gender	Female (ref)	-		-
	Male	0.06	-0.39–0.51	0.81
Group	Intervention (ref)	-		-
	Control	-0.14	-0.65–0.37	0.61
Time	T0 (ref)	-		-
	T1	0.85	0.47–1.23	< 0.0001
	T2	1.32	0.93–1.72	< 0.0001
	T3	2.11	1.7–2.52	< 0.0001
Group * Time	T1*Intervention (ref)	-		-
	T1*Control	0.12	-0.39–0.63	0.65
	T2*Intervention (ref)	-		-
	T2*Control	0.089	-0.43–0.6	0.74
	T3*Intervention (ref)	-		-
	T3*Control	-0.17	-0.72–0.38	0.54

CI: Confidence interval, SMS: Self-management support, T0: Baseline, T1: *i*mmediately after intervention, T2: Mid-point (after 3 months), T3: End-point (after 6 months).

#### Intervention fidelity

Nineteen interns (8 in the intervention group and 11 in the control group) were also evaluated on the BAP checklist a few weeks after receiving the intervention. The eight interns in the intervention group achieved 67% of the BAP items, while the 11 interns in the control group achieved 57% of the BAP items. Although 10 interns were evaluated for a second time, there was no significant improvement.

### Patients

#### Recruitment

Between March 2018 and April 2019, 42 patients agreed to participate in the study. Of the 19 and 23 patients assigned to the intervention and control groups, respectively, 8 (42%) and 11 (48%) patients in each group withdrew from the study. Most drop-outs could not be reached or they did not have future appointments.

#### Retention rate

Overall, 10 (52.6%) and 12 (52.2%) patients in the intervention and control groups completed the questionnaires at 8 weeks (i.e., end of the study questionnaires), respectively.

### Mid-study nominal group technique output

Twelve participants who attended the NGT at the mid-point of the study (2 managers, 1 research coordinator, 4 clinicians, and 5 interns) identified 20 challenges related to the implementation of SMS at CMCC (S7 Appendix). The 4 most important challenges were: 1) low engagement of interns, with a small number of participating interns impacting on the number of participating patients; 2) a lack of clarity around the purpose of the study, its potential benefits, and the role of their clinicians in the study; 3) interns assigned to the control group did not receive any formal training on how to use the BAP and felt lost as a result; and 4) vagueness of instructions for participating interns regarding when to begin implementing SMS with patients.

The NGT participants and research team suggested solutions to overcome the aforementioned challenges that we aimed to apply in the second round of the study. The proposed challenges were: *Engagement of interns*: highlighting the value of self-management and integrating SMS into clinical quizzes; *Lack of knowledge of role and vagueness of instructions*: distributing documents with clear instructions about the study timeline and the role of all participants, and *Training–control group*: asking the interns in the control group to access the online BAP training.

### End of study interviews

At the end of the study, 27 interviews were conducted with participants (8 clinicians, 5 participating interns, 2 interns who dropped-out, 7 non-participating interns, and 5 patients). Challenges faced during the study included (S8 Appendix): 1) some interns reported being too busy to participate due to their daily clinical duties and having to prepare for the national board exams; 2) patients were either not interested in participating or not eligible; 3) interns did not have enough time to attend the BAP training or spend more time with patients to deliver the SMS; and 4) patients had difficulty executing the SMS weekly. The reasons that the interns provided for choosing not to participate in the study included lack of time, not having eligible patients, and lack of clarity regarding study commitments. Reasons for withdrawing from the study included family issues, lack of time, and lack of confidence to implement SMS.

The interviewed patients stated that they were involved in developing their treatment plan and were able to modify it. They also mentioned that the treatment plan was clear. Regarding the evaluation tools, all interviewees acknowledged that they did not face any difficulties in completing the tools, which were clear and not too long. Lastly, the patients listed several aspects of the study that they liked: 1) the concept of SMS; 2) being involved in the treatment plan; 3) care providers’ concerns about them; and 4) receiving feedback from the care providers. On the other hand, they did not like the strong focus on exercises and diet, and having to complete the questionnaires 3 times (S9 Appendix).

The main solutions proposed by interviewees to improve study recruitment, adherence and retention rates included making the SMS a part of the internship, changing the time of introducing the study to the interns, having more training on SMS, and making the study more structured. Furthermore, the patients interviewed preferred having more person-to-person follow-ups with care providers and adding the posture exercises to the SMS program.

## Discussion

Findings from this study show that the proposed KT intervention can increase clinicians’ use of SMS, which in turn may which in turn may improve patients’ adherence to clinical recommendations and optimise patient health outcomes [38]. To our knowledge, this is the first study of chiropractic interns designed to promote the use of SMS with individuals living with spine pain. Implementing the KT intervention may therefore help interns adopt SMS as an evidence-based practice [25,45].

Nonetheless, this pilot cluster implementation trial had some important challenges in recruitment, retention and adherence that should be considered and resolved prior to conducting a full trial. While the recruitment rate of clinicians was high, only 25.6% of the 254 interns agreed to participate, and only 45 patients could be enrolled in the study. Further, only 5 clinicians in the control group, and 15 and 23 interns in the intervention and control groups, respectively returned the end of study questionnaires.

Poor recruitment and retention rates can impact the feasibility, validity, quality, and power of a study [64–68]. Retention is an important aspect of recruitment, and therefore the study design and the recruitment process need to be thoughtfully considered to avoid such recruitment challenges [69]. Poor retention can increase the cost of the study from a monetary and/or time perspective [68]. Also, adherence to the intervention plays a critical role in implementation research studies. Poor adherence affects the study’s validity and power, makes the study’s results less generalizable and may increase the cost and the duration of the study [70–72].

### Recruitment barriers

#### Clinicians

Our high recruitment rate (84%) of CMCC supervisory clinicians contrasts with the low recruitment rate of clinicians observed in field practice [73–75]. Clinicians working in teaching institutions may be more research oriented and interested in SMS and its effectiveness for patients. While a detailed invitation letter was sent to the supervisory clinicians explaining the study procedures at the study mid-point, the participating clinicians asked to receive additional details. Thus, a more detailed document was sent 6 months after the beginning of the study that outlined clinicians’ roles in the study (S10 Appendix), the study benefits and timeline.

#### Interns

In contrast, the recruitment rate for interns was low (25.6%). Challenges in recruiting university students in cohort studies and RCTs is in part due to the need for participants to be involved for longer periods of time [68]. Logistical factors such as lack of time, conflicting schedules, and starting the recruitment during a busy academic year may also reduce the recruitment rate [66,67,76,77]. Furthermore, unclear study instructions and procedures may have resulted in increasing the drop-out rate among participants [69].

The current study was introduced to the interns during the last year of their program, when they regularly see patients, and thus, are busy with clinical duties and preparing for the licensing exam. In addition, some interns stated that they lacked confidence to implement a new concept with patients as they did not yet have adequate clinical experience. Furthermore, we did not have a full-time research coordinator available at each of the three participating sites; this limited the communication between the researchers and the interns and likely further negatively impacted on the recruitment and retention rates. Nonetheless, interns at CMCC generally showed an interest in SMS.

Social exchange and self-determination theories may be helpful in shedding light on what motivates student participation in research. Social exchange theory postulates that when having to decide about participating in research, people tend to consider the many possible benefits [69,78,79] to themselves. Self-determination theory suggests that intrinsic (e.g., interesting project) and/or extrinsic factors may explain why students are motivated to participate in the study or not [69,80,81]. Although it is unclear what specific motivating factors were associated with participation in the study, offering a certificate on SMS, making the SMS a part of the internship and offering gift cards may improve the recruitment of interns.

#### Patients

Patient recruitment was low in large part due to low recruitment rate of interns who were responsible for implementing the SMS with patients. In addition, many spine pain patients attending CMCC outpatient clinics did not meet the eligibility criteria; age and inability to read and hold a conversation in English. While we started recruiting patients older than 65 in the last 6 months of the study, this did not improve recruitment. Lastly, some patients preferred receiving passive treatments and opted not to participate in the study.

### Retention rate

#### Clinicians

The retention rate of intervention group clinicians was 100% compared to 55% among those in the control group. One clinician in the control group dropped out from the study because none of his interns participated, and others might have lost interest because they did not receive any formal training in the BAP. Challenges that may lead to low retention rates included lack of time, poor explanation of the study, high study demands, and long study duration [69,77,82,83]. A recent pilot implementation trial among Canadian chiropractors also had low retention rates in both the intervention and control groups [73]. Additional barriers were lack of resources and clinicians’ fear of questions asked outside of their training [73,82].

#### Interns

The retention rate for interns in both groups did not reach our target goal of 80%. Some interns stated that they did not complete the study because of lack of time and being busy with internship duties. Also, the difficulties in recruiting patients for the study (e.g., lack of patient interest or availability and not meeting eligibility criteria due to their age or difficulty in communicating in English) resulted in some interns deciding to withdraw from the study.

#### Patients

Similarly, the retention rate for patients in both groups did not reach our target rate of 80%. The high drop-out rate of interns impacted the patient retention rate. The literature highlights barriers that might restrict patient retention: financial issues, job stress, and inadequate information from the care providers and family support [73,84,85].

### Adherence to the KT intervention

#### Clinicians

The adherence rate to all components of the KT intervention was 100%. This suggests that participating clinicians were interested in the study and in the intervention components, possibly in part because they were informed that they would receive a BAP certificate if they adhered to all study procedures. While the actual implementation of BAP was not assessed, the self-administered surveys showed that the clinicians improved in their skills and confidence to implement SMS. This improvement could reflect on the clinical practice of clinicians with their patients in general.

#### Interns

The high attrition rate among interns affected the adherence rate assessed over 6 months. The adherence rate would be higher if we consider only those interns who maintained their participation in the study.

### Proposed solution

Clinicians and interns made several helpful suggestions to improve the recruitment, retention, and adherence rates among interns for a full trial. Documentation should provide the study objectives, potential benefits of using SMS, roles of all participants, clear study instructions and a study timeline. An onsite full-time research coordinator should be available to help improve the recruitment of interns, provide needed information about the study procedures, and give ongoing feedback on the study process allowing to make regular adjustments. Also, concepts related to SMS should be formally introduced earlier in the chiropractic curriculum and built in as a core competency with clear goals and regular evaluations by supervising clinicians throughout the internship. Certifying the interns on BAP or other SMS strategies would encourage them to participate and adhere to the intervention components and serve them throughout their career. Lastly, offering incentives such as a certification and gift cards, and sensitizing interns to their roles as scholarly practitioners may motivate the interns and their patients to participate in, and adhere to the study’s procedures.

### Effect of KT intervention

The result of this study showed that the proposed intervention was effective immediately after its implementation on supervising clinicians’ perceived skills, self-efficacy and perceived importance related to the BAP; this is consistent with high-quality reviews having shown that providing educational workshops and materials to health professionals has a significant effect on improving their practice [86–88]. Regarding the interns, the results showed an improvement overtime of their perceived skills, self-efficacy and perceived importance related to the BAP. However, the results is not trustful as the small number of participating interns.

### Deviations from the project’s proposal

There were three deviations from the original approved protocol. First, the protocol indicated that we would recruit subjects from five clinics, but our manuscript reports only three participating chiropractic clinics because no research coordinator was available at the other two sites.

Second, the protocol indicated that the end-point of outcome assessment was three month while we reported results at six months. As the internship of one year duration are composed of two rounds, each round lasting six months, we assessed the feasibility of conducting the study at the end of each round (i.e. six months).

Third, we initially proposed using a stratified randomization based on the physical location of the PMTs. However, due to logistical issues at the participating clinics, clinicians and interns were assigned to one of two groups based on their working days.

## Limitations

Due to the high drop-out rate, we were unable to estimate some key parameters such as effect sizes for a future full-scale Randomised Clinical Trial (RCT), patient adherence to the recommended SMS approach for spine pain, and the effect of SMS intervention on the patients. In addition, the results of this study are not generalizable and may not apply to other participant groups in the same context.

## Potential bias and imprecision

A possible selection bias relates to the fact that it was not possible to randomize students or clinicians. Instead, they were assigned based on their workdays. Differences observed between patients in the control vs intervention groups may be due to information bias / misclassification (e.g. patient receiving the wrong diagnosis: acute vs chronic LBP or had a specific cause of back pain). Lastly, we did not control for interns who may have received the same information from another source such as a classroom (contamination).

The small sample size of interns could be a cause of imprecision regarding the effect of KT intervention on interns’ perceived skills, self-efficacy and perceived importance related to the BAP.

## Conclusion

This study provides new knowledge on the potential impact of a tailored KT intervention, and the factors that influence guideline implementation in clinical teaching environments. The recruitment of patients and interns can be challenging. However, the results of this pilot trial suggest that conducting a multi-site implementation trial may be feasible considering the identified barriers and related solutions. In addition, preliminary results suggest that the complex KT interventions can improve the SMS skills among clinicians and chiropractic interns.

## Supporting information

S1 Checklist CONSORT 2010 checklist of information to include when reporting a pilot or feasibility trial*. (DOC)

S1 Appendix “Brief action planning flow chart”. It provides the components and steps of the brief action planning model.
(DOCX)

S2 Appendix **A.** “Brief Action Planning (BAP) Tool Experience”. Questionnaire used to assess the clinicians’/interns’ perceived importance and confidence related to BAP. **B.** “Brief Action Planning Skills Survey”. Questionnaire used to assess the clinicians’/interns’ perceived skills related to BAP.
(DOCX)

S3 Appendix “BAP skills checklist (completed by observer during training)”. (DOCX)

S4 Appendix “Outcome measures”. It provides the description of study’s outcomes and their measures.
(DOCX)

S5 Appendix “Criteria to assess feasibility”. It provides the criteria used to assess the feasibility to conduct the larger implementation trial.
(DOCX)

S6 Appendix “Statistical analyses syntax”. It provides the statistical analyses syntax used in the quantitative analysis.
(DOCX)

S7 Appendix “Challenges related to the implementation of SMS at CMCC (output of NGT at the mid-point of the study)”. It provides detailed information about the challenges of study implementation that were raised up during the nominal group technique (NGT) at the mid-point of the study.
(DOCX)

S8 Appendix “Results of the individual interview with clinicians/interns at the end of the study”. It provides detailed information about the study that were raised up during the individual interviews with clinicians and interns at the end of the study: Challenges and suggestion.
(DOCX)

S9 Appendix “Results of the individual interview with patients at the end of the study”. It provides detailed information about the study that were raised up during the individual interviews with patients at the end of the study: Challenges and suggestion.
(DOCX)

S10 Appendix “Tasks of participating clinicians”. It provides the summary of supervisory clinicians’ tasks in the study.
(DOCX)

S1 Data (XLSX)

S2 Data (XLSX)

S3 Data (XLSX)

## Abbreviations

BAP: Brief action planning
BQ: Bournemouth Questionnaire
CI: Confidence Interval
CMCC: Canadian Memorial Chiropractic College
CPGs: Clinical practice guidelines
EBP: Evidence-based practice
KT: Knowledge translation
NGT: Nominal Group Technique
NRS: Numerical Rating Scale
MD: Mean Difference
PAM: Patient activation measure
PMT: Patient Management Teams
SMS: Self-management strategy
TDF: Theoretical domain framework
