# Supplementary material for: Promoting the use of a self-management strategy among novice chiropractors treating individuals with spine pain: A mixed methods pilot clustered-clinical trial
Source: PLoS One. 2022 Jan 21;17(1):e0262825. doi: 10.1371/journal.pone.0262825 (PMC8782363; doi:10.1371/journal.pone.0262825)
Supplement: S1 Appendix — It provides the components and steps of the brief action planning model. (DOCX) [file pone.0262825.s002.docx]

**S1 Appendix: Brief Action Planning Flow Chart**

(1) 1. Steven Cole DG, Connie Davis, Kathy Reims. Brief Action Planning Flow Chart: Centre for Collaboration, Motivation and Innovation (CCMI);; 2016 [cited 2022 8/1]. Available from: <https://centrecmi.ca/wp-content/uploads/2018/11/BAP_flow_Chart_2016-08-08.pdf>.
