# Supplementary material for: Promoting the use of a self-management strategy among novice chiropractors treating individuals with spine pain: A mixed methods pilot clustered-clinical trial
Source: PLoS One. 2022 Jan 21;17(1):e0262825. doi: 10.1371/journal.pone.0262825 (PMC8782363; doi:10.1371/journal.pone.0262825)
Supplement: S2 Appendix — A. “Brief Action Planning (BAP) Tool Experience”. Questionnaire used to assess the clinicians’/interns’ perceived importance and confidence related to BAP. B. “Brief Action Planning Skills Survey”. Questionnaire used to assess the clinicians’/interns’ perceived skills related to BAP. (DOCX) [file pone.0262825.s003.docx]

**S2A Appendix: Brief Action Planning (BAP) Tool Experience**

1. **How important is it for you to explain things in a way your patients can understand?**

0 1 2 3 4 5 6 7 8 9 10

Not at all important Extremely important

- **How confident are you that you can explain things in a way that your patients can understand?**

0 1 2 3 4 5 6 7 8 9 10

Not at all confident Extremely confident

1. **How important is it for you to elicit your patients’ preferences and cultural traditions when planning their care?**

0 1 2 3 4 5 6 7 8 9 10

Not at all important Extremely important

- **How confident are you that you can elicit your patients’ preferences and cultural traditions when planning their care?**

0 1 2 3 4 5 6 7 8 9 10

Not at all confident Extremely confident

1. **How important is it for you to work *collaboratively* with your patients to set goals to help them to improve their health and well being?**

0 1 2 3 4 5 6 7 8 9 10

Not important Very important

- - - - **How confident are you that you can work *collaboratively* with your patients to set goals to help them to improve their health and well being?**

0 1 2 3 4 5 6 7 8 9 10

Not at all confident Extremely confident

1. **How important is it for you to address the barriers that your patients’ face in setting or reaching their goals?**

0 1 2 3 4 5 6 7 8 9 10

Not at all important Extremely important

- **How confident are you that you can address the barriers that your patients’ face in setting or reaching their goals?**

0 1 2 3 4 5 6 7 8 9 10

Not at all confident Extremely confident

1. **How important is it for you to arrange or provide follow up with your patients regarding their goals?**

0 1 2 3 4 5 6 7 8 9 10

Not at all important Extremely important

- **How confident are you that you can arrange or provide follow up with your patients regarding their goals?**

0 1 2 3 4 5 6 7 8 9 10

Not at all confident Extremely confident

1. **How important is it for you to talk with your patients about including family or other supporters in a care plan?**

0 1 2 3 4 5 6 7 8 9 10

Not at all important Extremely important

- **How confident are you that you can talk with your patients about including family or other supporters in a care plan?**

0 1 2 3 4 5 6 7 8 9 10

Not at all confident Extremely confident

1. **How important is it for you to work with your patients to facilitate behavior changes that will make their health better?**

0 1 2 3 4 5 6 7 8 9 10

Not at all important Extremely important

- **How confident are you that you can work with your patients to facilitate behavior changes that will make their health better?**

0 1 2 3 4 5 6 7 8 9 10

Not at all confident Extremely confident

**S2B Appendix: Brief Action Planning Skills Survey**

Please check the box with **✓** that best describes the skills covered in any training you have had (including the recent BAP training) and your experiences with those skills. The responses range from left to right, starting with not having an opportunity to learn about it, to being confident using a skill you have learned. You may choose one answer per row.

| **I don’t know much about this** | **I tried to practice this during my training** | **I use this skill in my work** | **I am confident using this skill routinely in my work** |  |
| --- | --- | --- | --- | --- |
| The spirit of Motivational Interviewing |  |  |  |  |
| Teach-back for health literacy |  |  |  |  |
| Using reflections to emphasize hope and encourage change |  |  |  |  |
| Helping patients create action plans |  |  |  |  |
| Collaborative problem solving |  |  |  |  |
| Checking in on action plans |  |  |  |  |
| Using a confidence scale or ruler |  |  |  |  |
| Helping patients to talk about change |  |  |  |  |
| Developing strategies for working with challenging situations and people |  |  |  |  |
| Using Ask-Tell-Ask when giving information or advice |  |  |  |  |
