# Supplementary material for: Promoting the use of a self-management strategy among novice chiropractors treating individuals with spine pain: A mixed methods pilot clustered-clinical trial
Source: PLoS One. 2022 Jan 21;17(1):e0262825. doi: 10.1371/journal.pone.0262825 (PMC8782363; doi:10.1371/journal.pone.0262825)
Supplement: S3 Appendix — (DOCX) [file pone.0262825.s004.docx]

**S3 Appendix: BAP skills checklist (completed by observer during training)**

(2) Centre for Collaboration MaIC. Brief Action Planning Skills Checklist: Centre for Collaboration, Motivation and Innovation (CCMI),; 2018 [cited 2022 08/01]. Available from: <https://centrecmi.ca/wp-content/uploads/2018/05/BAP_Skills_Checklist_2018-05-01.pdf>.
