# Supplementary material for: Promoting the use of a self-management strategy among novice chiropractors treating individuals with spine pain: A mixed methods pilot clustered-clinical trial
Source: PLoS One. 2022 Jan 21;17(1):e0262825. doi: 10.1371/journal.pone.0262825 (PMC8782363; doi:10.1371/journal.pone.0262825)
Supplement: S4 Appendix — It provides the description of study’s outcomes and their measures. (DOCX) [file pone.0262825.s005.docx]

S4 Appendix: Outcome measures

| **Outcome** | **Source** | **Description of measures** | **Data collection time points** | |
| --- | --- | --- | --- | --- |
| **Feasibility** | | | | |
| Recruitment | Clinicians and Interns | Measured as a proportion of clinicians and interns potentially eligible for participating.  Participation/recruitment rate = number of clinicians and interns agreeing to participate divided by number of eligible interns and patients. | Initial Stage | |
| Adherence  to protocol | Clinicians and Interns | For those randomized to intervention arm, measured through the rates of attendance of the online training, practice and feedback session, and workshop training | Within 3 months | |
| Retention | Clinicians, Interns and patients | Retention rate = number of clinicians, interns or patients who completed follow-up of all outcome measures at 6 month (clinicians, intern)/ 2 month (patient) divided by number of clinicians, interns or patients who were randomized. |  |  |
|  | Clinicians and Interns | Rate of completion of clinicians and interns encounter forms and questionnaires including the BAP and levels of knowledge and self-efficacy. | Baseline | 6 months |
|  | Patients | Rate of completion of patient encounter forms and questionnaires including the BAP and PAM, NRS, Bournemouth Questionnaire, quality of life (PROMIS Global Health Questionnaire), and satisfaction | Baseline | 2 months |
