# Supplementary material for: Promoting the use of a self-management strategy among novice chiropractors treating individuals with spine pain: A mixed methods pilot clustered-clinical trial
Source: PLoS One. 2022 Jan 21;17(1):e0262825. doi: 10.1371/journal.pone.0262825 (PMC8782363; doi:10.1371/journal.pone.0262825)
Supplement: S5 Appendix — It provides the criteria used to assess the feasibility to conduct the larger implementation trial. (DOCX) [file pone.0262825.s006.docx]

**S5 Appendix: Criteria to assess feasibility**

| **Construct** | **Eligibility proportion** |
| --- | --- |
| **Clinicians and interns** | |
| Recruitment | Trial acceptance rate: > 55% agree to participate within 6 weeks.  Target population = 73 (assuming an 80% retention rate) |
| Adherence to protocol | >90% of participants will complete all components of the KT intervention (BAP webinar and self-management learning module and 2 clinical vignettes, and coaching by BAP certified supervisory clinicians). |
| Retention | 80% of participants will complete 6 months of follow-up |
| **Patients** | |
| Retention | > 80% will complete patient encounter forms (BAP survey, Patient Activation Measure (PAM), NRS, Bournemouth Questionnaire, quality of life (PROMIS Global Health Questionnaire), and satisfaction) with and follow-up at 8 weeks. |
