# Supplementary material for: Promoting the use of a self-management strategy among novice chiropractors treating individuals with spine pain: A mixed methods pilot clustered-clinical trial
Source: PLoS One. 2022 Jan 21;17(1):e0262825. doi: 10.1371/journal.pone.0262825 (PMC8782363; doi:10.1371/journal.pone.0262825)
Supplement: S6 Appendix — It provides the statistical analyses syntax used in the quantitative analysis. (DOCX) [file pone.0262825.s007.docx]

**S6 Appendix: Statistical analyses syntax**

**Wilcoxon-Mann-Whitney test**

proc npar1way data = PHASE2_ALLTIME_DIF_CLIN1 wilcoxon;

class InterventionG;

var SM_IMPORTANT_DIF01 SM_CONFIDENT_DIF01 BAP_Skills_DIF01;

run;

proc npar1way data = PHASE2_ALLTIME_DIF_CLIN1try wilcoxon;

class InterventionG;

var SM_IMPORTANT_DIF03 SM_CONFIDENT_DIF03 BAP_Skills_DIF03;

run;

**Wilcoxon signed rank sum test**

proc univariate data = ALL_DATA_CLINICIANS;

var BAP_Skills_DIF01 BAP_Skills_DIF02 BAP_Skills_DIF03 sm_confident_dif01 sm_confident_dif02 sm_confident_dif03

SM_IMPORTANT_DIF01 SM_IMPORTANT_DIF02 SM_IMPORTANT_DIF03;

by InterventionG;

run;

**Mixed-effects Models**

PROC MIXED data= Intern_Datanew3 covtest noclprint method= ML;

class ID time1 (ref = '0') Clinician_ID gender Group_SMS;

model BAP_Skills = Time1 age gender Group_SMS time1*Group_SMS/ solution ddfm = SATTERTHWAITE;

random intercept Time1 / sub=ID(Clinician_ID) type=vc;

lsmeans time1*Group_SMS;

run;

PROC MIXED data= INTERNT_BOTHEXP3 covtest noclprint method= ML;

class ID2 time1 (ref = '0') Clinician_ID gender Group_SM;

model SM_IMPORTANT0 = Time1 age gender Group_SM time1*Group_SM/ solution ddfm = SATTERTHWAITE;

random intercept Time1 / sub=ID2(Clinician_ID) type=vc;

lsmeans time1*Group_SM;

run;

PROC MIXED data= INTERNT_BOTHEXP3 covtest noclprint method= ML;

class ID2 time1 (ref = '0') Clinician_ID gender Group_SM;

model SM_CONFIDENT0 = Time1 age gender Group_SM time1*Group_SM/ solution ddfm = SATTERTHWAITE;

random intercept Time1 / sub=ID2(Clinician_ID) type=vc;

lsmeans time1*Group_SM;

run;
