# Supplementary material for: Promoting the use of a self-management strategy among novice chiropractors treating individuals with spine pain: A mixed methods pilot clustered-clinical trial
Source: PLoS One. 2022 Jan 21;17(1):e0262825. doi: 10.1371/journal.pone.0262825 (PMC8782363; doi:10.1371/journal.pone.0262825)
Supplement: S7 Appendix — It provides detailed information about the challenges of study implementation that were raised up during the nominal group technique (NGT) at the mid-point of the study. (DOCX) [file pone.0262825.s008.docx]

**S7 Appendix: Challenges related to the implementation of SMS at CMCC (output of NGT at the mid-point of the study)**

| **Challenges** | Details |
| --- | --- |
| Time of start of study *(6 considered it important)* | The interns in this study were recruited in November. To increase the acceptance rate, it would be better to start recruiting interns in May of each year where the new interns start the first rotation of internship. Itis easier to start the study earlier in the beginning of clinic year or even before.  Nine participants agreed and 6 participants considered this point as an important challenge.  A clinician prefers to have a short time frame.  An intern mentioned that it was busy at the beginning of the clinic year, and she would have not participated if she was invited at that time. It also depends on the clinic site. Some clinics were too busy and interns could not commit to participate in the study. |
| - Low # of participating interns *(8 considered it important)* - Interns recruitment *(10 considered it important)* - Engagement of interns *(10 considered it important)* | Low # of participating interns resulted in the low number of participating patients since one of the patients’ inclusion criteria was (patients who are treated by a consenting chiropractic intern).  **Solution**: Having clear instructions and schedule will increase the # of participating interns.  A clinician thinks that interns who have a good communication skills will participate in the study while who do not have these skills will not participate (she considered that a selection bias). Therefore, we need to encage more interns to participate. We may use incentives to the interns (gift card).  Another way to engage interns is to introduce and convince the interns on the value of self-management and integrate SMS into the clinical quizzes. |
| Training too long for clinicians and interns *(one considered it important)* | The process of BAP certification took long time. Clinicians would have preferred to have less steps to be certified.  The workshop was helpful for clinicians.  Two interns did not find the online modules were helpful, and the training with clinician and peers was more useful.  Also, the interns needed to wait for time to be trained and start implement SMS with patients. |
| Length of SMS intervention *(no one considered it important)* | The SMS session (interaction with patient) was supposed to be 5 minutes, and it was never 5 minutes; it’s longer. 2 interns dropped out from the study because of that. SMS is not a routine practice for them so it is not easy to get used to the skills and make the session shorter. Experience and practice on SMS will decrease the length of SMS intervention. However, one intern mentioned that her SMS session was fast and short.  Documentation of SMS was slow. Thus, training on documentation will be useful. |
| Lack of focus/ readiness *(5 considered it important)* | Clinicians are busy with other stuff, and they have other clinical stuff which are more prioritized. |
| Consistency of training *(no one considered it important)* | 2 groups of clinicians attended the telephone practice (the first call), one group complained about the trainer since s/he was asking about the other trainer! However, the clinicians liked the certification telephone training (the one took place in February 2018). |
| Timing of training *(no one considered it important)* | There is a mismatch between learning SMS as an intervention and the curriculum. The SMS training came late and the students were asked to learn new intervention at clinic, which was difficult. A solution was proposed to introduce SMS to the students in the third year. Interns preferred to have the training earlier. |
| Lack of knowledge of role *(9 considered it important)* | The role of clinicians in the study was not clear. In addition, the purpose of the study and the benefit of it needed to be clear. Clear instructions document is needed. |
| - Organization – Surveys *(no one considered it important)* - Vagueness of instructions (recruitment, survey, intervention) *(6 considered it important)* | An intern found there were a lot of requirements, and its better to have a schedule of the tasks.  It was not clear for interns when they supposed to start the SMS implementation with patients  The timeline of the study needs to be added to the students’ presentation slides |
| Training – control group *(8 considered it important)* | No training, people in control group felt lost. The intern understood the concept of SMS but did not know enough to execute it. |
| Literacy, language, and age restriction (exclusion criteria) *(no one considered it important)* | Some patients do bit speak English so they couldn’t participate in the study. However, this was an exclusion criterion.  A significant number of patients at CMCC are older than 65 years and because of that they were not invited to the study |
| - Patient response *(one considered it important)* - Patient recruitment – scheduling *(no one considered it important)* | Interns could not start the implementation of SMS until patients complete the baseline questionnaire, and this took time  A patient did not participate in the study because she did not know the survey schedule.  A solution is to provide patients with schedule to fill out the surveys. Having a research assistant will help,, and it will also help in the organization issues.  Increase number of participating interns will also increase number of participating patients |
| F/U engagement of patient in SMS *(no one considered it important)* | It was difficult to keep patients interested in the SMS because the interns feel that they are repeating same thing over time. It was proposed to have different scripts for follow-up sessions |
| Technical challenges *(2 considered it important)* | Survey links: some participants received a message that they already completed the survey but they actually did not.  Online training: certain internet explorer (Firefox) needed to have access to the online training. |
| - Distance of principle investigator and research team *(5 considered it important)* - Response time from research team *(one considered it important)* | There is no permanent research assistant. If there is more research staff it will increase the engagement of participants.  Sometimes it takes time to hear from the research team. |
