# Supplementary material for: Promoting the use of a self-management strategy among novice chiropractors treating individuals with spine pain: A mixed methods pilot clustered-clinical trial
Source: PLoS One. 2022 Jan 21;17(1):e0262825. doi: 10.1371/journal.pone.0262825 (PMC8782363; doi:10.1371/journal.pone.0262825)
Supplement: S8 Appendix — It provides detailed information about the study that were raised up during the individual interviews with clinicians and interns at the end of the study: Challenges and suggestion. (DOCX) [file pone.0262825.s009.docx]

**S8 Appendix: Results of the individual interview with clinicians/interns at the end of the study**

|  | Response |
| --- | --- |
| Challenges | **Clinicians:**   - Patient recruitment (they were not interested or not eligible, or the clinicians were busy with other tasks) (5) - The assessment of patients’ compliance with SMS was difficult (2) - Interns recruitment: timing issue (2); interns were busy and overwhelmed with daily clinical duties and board exams/ no eligible patients - It was difficult to train the intern because of: lack of time (2)/ clinicians needed more practice before train the intern/ interns were not familiar with SMS - It was difficult to make sure that interns follow the plan - There was a gap between clinicians’ training and intern recruitment, thus it wasn’t comfortable to evaluate the interns on SMS (solution: having a supervisor to evaluate clinicians on SMS implementation and this will enable them to evaluate interns) - Remembering the practice of SMS   **Interns:**   - Patient recruitment (they were not interested or not eligible) (5) - Lack of time (4) - Having a structured script   *patients didn’t receive information about SMS and its benefits |
| Receiving information about the study | **Clinicians:**   - All information about the study was received and it was clear and outlined (7)   **Interns**   - All information about the study was received and it was clear (7)/ Information on the objective and rules was not received / information about the procedures wasn’t clear - It wasn’t clear how many patients should be recruited - It was difficult to recruit patients as they didn’t want to spend time in filling out the documents |
| Intervention content | **Clinicians:**   - Intervention contents were clear (8) - It was easy to access the online training (6) - The intervention was useful to deliver SMS with patients (6) - Flowchart and online training were enough for introductory and moderate levels/ online training was enough to implement SMS - The repetition of information was useful   **Interns**   - Intervention contents were clear (4) - It would be better to have in-person training/ more training - It was easy to access the online training (3)/ I didn’t access the online training (3) - No meeting with the supervisory clinician (busy clinic)/ the training session with the clinician was helpful - The intervention was useful to deliver SMS with patients (6) - The intervention was enough to implement SMS/ online training alone is not enough to deliver SMS. - Scripted intervention was not good, natural conversation was preferred |
| Supportive handout (BAP flowchart, patients documents) | **Clinicians:**   - Documents for patients were received (5)/ supportive documents were not received (2) - The supportive documents helped to implement SMS (5) - It was easy to train the **interns**, especially they were introduced to self-management / interns’ training was initially easy, however, continuing the practice of SMS was difficult.   **Interns**   - The supportive documents were received (6)/ documents for patients were received - The supportive documents helped to implement SMS (6) / The supportive documents didn’t help to implement SMS |
| Study process | **Clinicians:**   - It was easy to understand the study’s documents (7) - Duties regarding the deal with interns were not clear (1)   **Interns:**   - It was easy to understand the study’s documents (6) - The surveys were clear and not too long (6) |
| Support from clinic | **Clinicians:**   - Clinicians were supported by the clinic and got quick answers for their questions (7) - Talking to colleagues and research team about the challenges (4)*/ not talking to others about the challenges (3)   *We cannot force patients to accept the active treatment  **Interns:**   - Interns were supported by the clinic (6)/ wasn’t supported as the supervisory clinician and interns were busy. - Interns got quick answers for their questions (6) - Talking to supervisory clinicians and research team about the challenges (5)/ Not talking to others about the challenges |
| Suggestion | **Clinicians:**   - Certify the interns on SMS - Making the SMS a part of the internship (as a credit or an assignment) (4) - Introducing the SMS to the interns during the 2^nd^ or 3^rd^ year (3) / introducing the SMS to the interns through half of the year as they may get comfortable with clinical procedures / introducing the SMS to the interns through half of the 1^st^ rotation as they may get comfortable with clinical procedures - Using app or web-page to help patients download supportive documents and they can be tracked by clinicians - Showing the interns where SMS fits in the clinical internship - Having an RA to remind clinicians to implement SMS with patients - Compensation for interns   **Interns:**   - Providing in-person training / having more training - Providing interviewing skills session - Making the study more structured (how to implement SMS/ how to follow-up, and how to use the flowchart) - Asking the clinicians and research team to help in patient recruitment - Introducing the SMS to the interns at the end of 3^rd^ year/ introducing the study at the beginning of the internship (3) - Having a natural conversation with patients rather than scripted conversation - Making the implementation of SMS mandatory |
| Reasons of drop-out (Interns) | - Family reason (interested in SMS) - Lack of time - Lack of confidence |
