# Supplementary material for: Promoting the use of a self-management strategy among novice chiropractors treating individuals with spine pain: A mixed methods pilot clustered-clinical trial
Source: PLoS One. 2022 Jan 21;17(1):e0262825. doi: 10.1371/journal.pone.0262825 (PMC8782363; doi:10.1371/journal.pone.0262825)
Supplement: S9 Appendix — It provides detailed information about the study that were raised up during the individual interviews with patients at the end of the study: Challenges and suggestion. (DOCX) [file pone.0262825.s010.docx]

**S9 Appendix: Results of the individual interview with patients at the end of the study**

|  | Response |
| --- | --- |
| Challenges | - Performing the exercise weekly (1)  - The adherence to SMS because of pain (1)  - Exchanging the health information with the care providers (1) |
| Receiving information about the study | - receiving enough information about the study (3)/ not receiving (2)  - the information was clear (2) |
| Self-management and making plan with care provider | - involving in the making of treatment plan (5)  - treatment plan was clear (5)  - implementing the treatment plan with confident (1)  - able to adapt the exercise (4)  - spending enough time on SMS with care provider (2) / not spending enough time (1) |
| Intervention content including SMS handout, exercise sheet, and exercise website | - All information about the study was received and it was clear (4) / Information on the study was not received (1)  - The exercise sheet was received (3)  - The supportive documents were clear and helped to implement SMS (5) |
| Evaluation tools | - There were no difficulties to complete the questionnaires (5)  - The questionnaires were clear (5)  - The questionnaires were not long (5) |
| Support from clinic | - The patients were supported by the clinic and care providers (5)  - The patient had a phone call from care provider to check on the SMS plan (1)/ did not have (1)  - The patients got answers for the questions about the study and SMS (4) |
| Things in the study you like the most/ | - The concept of SMS (2)  - Involving in the treatment plan (1)/ Having the chance to set treatment goals and receiving help to achieve the goals (2)  - The clinic was supportive (1) and care providers concerned about us (2)  - Receiving feedback from the care providers (1) |
| The ones you didn’t like the most | - The program focuses only on exercises and diet (1)  - Filling out the questionnaires 3 times (1) |
| Suggestions | - More follow-up from the care provider (1)/ Having a person to person follow-up (1)  - Posture exercises should be added to the program (1)  - More clarity on how many times patients need to come to the clinic (1) |
