# Supplementary material for: Promoting the use of a self-management strategy among novice chiropractors treating individuals with spine pain: A mixed methods pilot clustered-clinical trial
Source: PLoS One. 2022 Jan 21;17(1):e0262825. doi: 10.1371/journal.pone.0262825 (PMC8782363; doi:10.1371/journal.pone.0262825)
Supplement: S10 Appendix — It provides the summary of supervisory clinicians’ tasks in the study. (DOCX) [file pone.0262825.s011.docx]

**S10 Appendix: Tasks of participating clinicians**

Dear,

Thank you for your interest in the CMCC Patient Self-management Strategy Study: SMILE Study. This document highlights the tasks of participating clinicians in this study unfolding between May and November 2018 (1^st^ rotation of the class of 2019):

- Attend the self-study webinar (50 minutes) and online educational module on the Brief Action Planning (BAP) (22 minutes).
- When: Week of May 28^th^
- During the 5 hours weekly administrative time of the internship, provide your interns with a demonstration of skills using BAP during clinical encounters with patients
- When: by mid-June
- Frequency: one time, *unless if more is needed*
- You DO NOT need to complete any survey for this rotation.
- Evaluate the participating interns their patient – intern interaction using BAP evaluation checklist (2 times)
- When: (1) Week of July 30^th^
- When: (2) Week of October 15^th^

If you have any questions, please contact Dr. Joyce Lee at CMCC clinic

Phone: 416 482 2340 ext. 266

Email: jlee@cmcc.ca

Thank you,

The research team
